# Effectiveness of Nurse-Led Cardiac Rehabilitation Compared to Routine Care: A Systematic Review

**DOI:** 10.7759/cureus.96408

**Published:** 2025-11-09

**Authors:** Naqib Miah, Amna Halili, Md Raqeebuzzaman

**Affiliations:** 1 Foundation Programme, Bedfordshire Hospitals National Health Service (NHS) Foundation Trust, Luton, GBR; 2 Foundation Programme, East and North Hertfordshire Teaching National Health Service (NHS) Trust, Hertfordshire, GBR; 3 Cardiology, Medical College for Women and Hospital, Dhaka, BGD

**Keywords:** adult cardiac surgery, cardiac function, cardiac risk factors and prevention, cardiopulmonary rehab, clinical outcomes, functional capacity, nurse-led interventions, quality of life (qol), routine, s: hospital readmissions

## Abstract

Nurse-led cardiac rehabilitation (CR) models place nurses at the centre of exercise training, risk factor modification and education. By leveraging nursing expertise and structured pathways, these services are designed to improve accessibility, adherence, and continuity while preserving safety and clinical standards. Despite increasing adoption, their impact compared to routine care remains uncertain. We therefore conducted a systematic review which evaluated the effectiveness of nurse-led CR compared with routine care for adults with cardiovascular disease.

This review was conducted and reported in accordance with PRISMA guidelines. The protocol was prospectively registered on PROSPERO (CRD420251039182). Comprehensive searches were performed across MEDLINE (PubMed and Ovid), Embase, Cochrane CENTRAL, CINAHL, and Scopus for studies published between January 2000 and June 2025. Randomised controlled trials (RCTs) comparing nurse-led CR, defined as programmes primarily delivered, coordinated, or supervised by nurses with routine or usual care, were included. Eligible participants were adults (≥18 years) with established cardiovascular disease. Studies led exclusively by non-nursing professionals or involving paediatric populations were excluded. Two reviewers independently screened studies, extracted data, and assessed risk of bias using the Cochrane RoB 2 tool. Certainty of evidence was appraised using the GRADE framework.

Of 1,997 identified records, six RCTs (n=922) met the inclusion criteria, conducted across India, China, and the UK. Populations encompassed stable cardiac cohorts, including heart failure and post-PCI patients. Interventions ranged from centre-based to hybrid and virtual formats. Nurse-led CR consistently produced significant, clinically meaningful improvements in quality of life (QoL) (moderate-certainty evidence), observed across both generic (SF-36, EuroQol) and disease-specific (MLHFQ, SAQ, MacNew) instruments, with benefits sustained up to seven months. Functional capacity also improved significantly (moderate-certainty evidence), reflected by increases in walking distance (e.g., +44.9 m on the 6-minute walk test), strength, and mobility. Patient-reported outcomes such as angina frequency and perceived exertion similarly favoured the intervention. Four studies demonstrated reductions in hospital readmissions (absolute risk reduction 8-10%) and fewer total hospital days (low-certainty evidence). Mortality was rarely reported, with no significant difference between groups over short-term follow-up (very low-certainty evidence).

Nurse-led cardiac rehabilitation is an effective and safe model of care that significantly enhances quality of life and functional capacity in adults with cardiovascular disease. Evidence suggests potential benefits in reducing hospital utilisation, warranting confirmation in larger, longer-term trials. The flexibility of nurse-led CR across diverse settings supports its scalability and integration into existing services, particularly in systems constrained by workforce shortages or serving underserved populations. Broader implementation could strengthen secondary prevention efforts and improve key patient-centred outcomes. Future research should incorporate standardised outcome measures and formal economic evaluations to guide policy and resource allocation.

## Introduction and background

Cardiovascular disease (CVD) continues to be the leading cause of death worldwide, accounting for approximately 17.9 million deaths annually and contributing to around one-third of all global mortality [1]. Beyond premature mortality, CVD is a major contributor to long-term morbidity, disability, and reduced quality of life, resulting in recurrent hospitalisations, productivity loss, and a significant financial burden on healthcare systems [2,3]. The rising prevalence of CVD, driven by ageing populations, sedentary lifestyles, and comorbidities such as diabetes and obesity, highlights the growing need for effective secondary prevention strategies to reduce disease progression and improve patient outcomes [3].

Cardiac rehabilitation (CR) is a cornerstone of secondary prevention for patients with established CVD. It is a structured, multidisciplinary intervention that integrates supervised exercise training, risk factor modification, education, and psychosocial support [4]. Substantial evidence demonstrates that CR reduces cardiovascular mortality, recurrent hospital admissions, and adverse cardiac events, while improving exercise tolerance, treatment adherence, and health-related quality of life (QoL) [5,6]. Consequently, it is strongly recommended by major international guidelines, including those of the American Heart Association (AHA), American College of Cardiology (ACC), and European Society of Cardiology (ESC), for patients following acute coronary syndromes and revascularisation procedures and in those with chronic heart failure [7,8].

Despite its well-documented benefits, participation in CR remains persistently low worldwide. Referral and completion rates often fall below 50%, with lower uptake among women, older adults, and individuals from socioeconomically deprived or rural backgrounds [9,10]. Barriers such as workforce shortages, limited programme capacity, travel distance, time constraints, and socioeconomic factors continue to limit accessibility. In low-resource settings, these challenges are compounded by a lack of specialised staff and infrastructure, further widening health inequalities [9,10].

To address these gaps, nurse-led models of care have emerged as a promising alternative or adjunct to traditional physician-led CR. Nurses play a vital role in chronic disease management through patient education, behavioural support, and continuity of care, which are key components of effective secondary prevention [11-13]. Evidence from nurse-led interventions in other chronic conditions, such as hypertension, diabetes, and heart failure, has shown comparable or improved outcomes relative to standard care, including better adherence to treatment, improved symptom control, and enhanced patient satisfaction [11-13].

Within the context of cardiac rehabilitation, nurse-led models have been proposed as a feasible and potentially cost-effective way to expand access without compromising quality or safety [14]. Randomised controlled trials (RCTs) have demonstrated improvements in functional capacity, QoL, and adherence to lifestyle modifications among patients managed in nurse-led CR settings [15,16]. However, existing studies vary considerably in design, duration, and outcome measures, making it difficult to draw firm conclusions.

Previous systematic reviews of CR have primarily evaluated multidisciplinary or physician-led programmes, often grouping nurse-led interventions within broader models of care [17-19]. The Cochrane overview of reviews by Anderson and Taylor [17] synthesised evidence from six Cochrane systematic reviews encompassing approximately 98,000 participants. It confirmed that exercise-based CR significantly improved QoL and reduced hospital readmissions but showed limited effects on mortality. That overview also highlighted considerable heterogeneity across trials and emphasised the need for research into “contemporary models of CR delivery”.

Building on this, the recent Cochrane update by McDonagh et al. [18] compared home-based versus centre-based CR across 24 randomised controlled trials involving more than 3,000 participants. The review demonstrated comparable clinical and QoL outcomes between the two delivery formats, supporting the wider implementation of professionally supervised home-based programmes to enhance accessibility and participation.

Although a systematic review has examined nurse-led CR specifically in post-coronary artery bypass graft (CABG) patients, reporting mixed effects on QoL [19], there remains no comprehensive synthesis evaluating the effectiveness of nurse-led CR across all cardiac disease populations. This represents a gap in the literature, particularly given that nurses frequently serve as the principal coordinators of exercise training, education, medication titration, and follow-up in both hospital- and community-based settings. The present review, therefore, addresses this gap by synthesising randomised controlled trials evaluating the effectiveness of nurse-led CR compared to routine care across diverse cardiac populations and delivery modalities.

This systematic review aims to evaluate and narratively synthesise the effectiveness of nurse-led cardiac rehabilitation compared with routine care in adults with cardiovascular disease, focusing on outcomes of quality of life, functional capacity, hospital readmissions, and mortality.

## Review

Methods

This systematic review was conducted in accordance with the Preferred Reporting Items for Systematic Reviews and Meta-Analyses (PRISMA) [20]. The protocol was registered on the International Prospective Register of Systematic Reviews (PROSPERO [CRD420251039182]; registered 17/05/2025).

Eligibility Criteria

Studies were selected according to predefined PICOS criteria. The population of interest was adults (≥18 years) with cardiovascular disease undergoing cardiac rehabilitation, including those with previous myocardial infarction, coronary artery bypass graft surgery, percutaneous coronary intervention, heart failure, or other forms of established cardiovascular disease. The intervention of interest was nurse-led cardiac rehabilitation (CR), defined as programmes primarily delivered, coordinated, or supervised by nurses. Eligible programmes included structured education, lifestyle counselling, medication optimisation, supervised or home-based exercise, and follow-up support delivered in-person, in hybrid formats, or virtually. The comparator was routine or usual care, defined as standard clinical management without a structured nurse-led CR programme, such as discharge advice or routine follow-up in primary or secondary care. To be considered nurse-led, programmes required nurses to act as the principal supervisors/coordinators or primary care providers, even when supported by multidisciplinary teams.

The prespecified outcomes of interest were quality of life (QoL), assessed using disease-specific or generic validated tools; functional capacity; hospital readmissions (all-cause or cardiovascular-related); and all-cause mortality. Although the protocol prespecified additional outcomes, including cardiovascular mortality, major adverse cardiovascular events, programme adherence, and cost-effectiveness, these were not reported in the included trials and therefore were not analysed. Only randomised controlled trials (RCTs) were eligible for inclusion. Studies were excluded if they were not RCTs, if they included paediatric or non-cardiac populations, or if interventions were led exclusively by other allied health professionals without nurse involvement. RCTs were selected because this review sought to determine the causal effectiveness of nurse-led cardiac rehabilitation compared with routine care. Including only RCTs minimised confounding and provided the highest level of evidence for intervention evaluation.

Search Strategy

We searched MEDLINE (via PubMed and Ovid), Embase, Cochrane Central Register of Controlled Trials (CENTRAL), and CINAHL, from 1 January 2000 to 30 June 2025. Search terms incorporated MeSH terms and free-text keywords relating to “cardiac rehabilitation” in combination with terms for “nurse-led care” and comparators such as “usual care” or “standard care”. To ensure compatibility, the search string was then adapted for the respective database. The complete PubMed search string and detailed strategies for all databases are provided in Table 1. Searches were limited to English language studies, and no grey literature was searched. 

**Table 1:** Search strategy used in the PubMed database [Mesh] = Medical Subject Headings; [tiab] = Title/Abstract field.

Search Line	Query
1	"Cardiac Rehabilitation"[Mesh] OR "cardiac rehab*"[tiab] OR "heart rehab*"[tiab] OR "secondary prevention"[tiab] OR "coronary artery disease"[tiab]
2	"Nurse-Led"[tiab] OR "nurse-managed"[tiab] OR "nurse-coordinated"[tiab] OR "nurse-run"[tiab] OR "Nurses"[Mesh] OR "Nursing"[Mesh] OR "Nurse's Role"[Mesh] OR ("nurse"[tiab] AND ("care"[tiab] OR "program"[tiab] OR "rehab*"[tiab] OR "intervention"[tiab])) OR ("nursing"[tiab] AND ("care"[tiab] OR "program"[tiab]))
3	("2000/01/01"[Date - Publication] : "2025/05/30"[Date - Publication])
Final Set	1 AND 2 AND 3

Study Selection

All studies were then imported into Rayyan to firstly remove duplicates. Two reviewers (NM and AH) then independently screened article titles, followed by their abstracts, against the pre-specified inclusion and exclusion criteria. Full-text articles were then assessed for inclusion. Any disagreements during screening were resolved through discussion or by consulting the third researcher (MR). This process was documented using a PRISMA flow diagram.

Data Extraction

Data were extracted independently by two reviewers (NM, AH) using a piloted, standardised form. For each included trial, we recorded study identifiers (year of publication, country), study design, and sample size. Participant characteristics were extracted alongside intervention details, including programme type, delivery mode (in-person, hybrid, or virtual), duration, and the outcomes we assessed.

For each outcome, we extracted the measurement tools used (for example, the Minnesota Living with Heart Failure Questionnaire, SF-36, incremental shuttle walk test, or 6-minute walk test), along with effect estimates, confidence intervals, and p-values (where reported). When outcomes were reported at several time points, we extracted data from the longest available follow-up within 12 months. In cases where results were displayed only in figures, values were approximated where feasible. Any disagreements in data extraction were resolved through discussion, with a third reviewer (MR) consulted if consensus was not reached.

Risk of Bias

Two reviewers (NM, AH) independently assessed risk of bias using the Cochrane Risk of Bias 2 (RoB 2) tool [21]. The following domains were evaluated: bias arising from the randomisation process, deviations from intended interventions, missing outcome data, outcome measurement, and selection of the reported result. Again, when consensus was not reached through discussion, the third researcher (MR) reconciled. Each outcome was judged as low risk, some concerns, or high risk of bias. Results were presented in graphical format.

Data Synthesis

Due to substantial clinical and methodological heterogeneity in interventions, outcome measures, and follow-up periods, a quantitative meta-analysis was deemed inappropriate. A narrative synthesis was therefore undertaken. Instead, findings were synthesised narratively, structured by outcome domain (quality of life, functional capacity, hospital readmissions, and mortality). Both the direction and magnitude of effects were considered, and where possible, results were interpreted in relation to established thresholds for clinical significance.

Certainty of Evidence

The certainty of the evidence for each outcome was assessed using the Grading of Recommendations Assessment, Development and Evaluation (GRADE) framework [22]. Two reviewers (NM and AH) independently performed the assessments, and any disagreements were resolved through discussion. The evaluation considered five domains: risk of bias, inconsistency, indirectness, imprecision, and publication bias.

Risk of bias was judged on the basis of the RoB 2 assessments conducted for each trial. Inconsistency was evaluated by examining variability in the direction and magnitude of effects across studies. Indirectness was considered by assessing how well the study populations, interventions, comparators, and outcomes matched the review question. Imprecision was evaluated by considering the sample sizes of the included studies and the width of confidence intervals around effect estimates. Publication bias was considered in relation to selective reporting or underreporting of negative findings.

Randomised controlled trials were initially rated as providing high-certainty evidence. The certainty rating for each outcome was downgraded by one or more levels if serious concerns were identified in any of the five domains. Final certainty ratings were categorised as high, moderate, low, or very low. Explanations for each downgrading decision were provided in the footnotes of the Summary of Findings table, which presents the effect estimates alongside the certainty of evidence ratings.

Results

**Figure 1:**
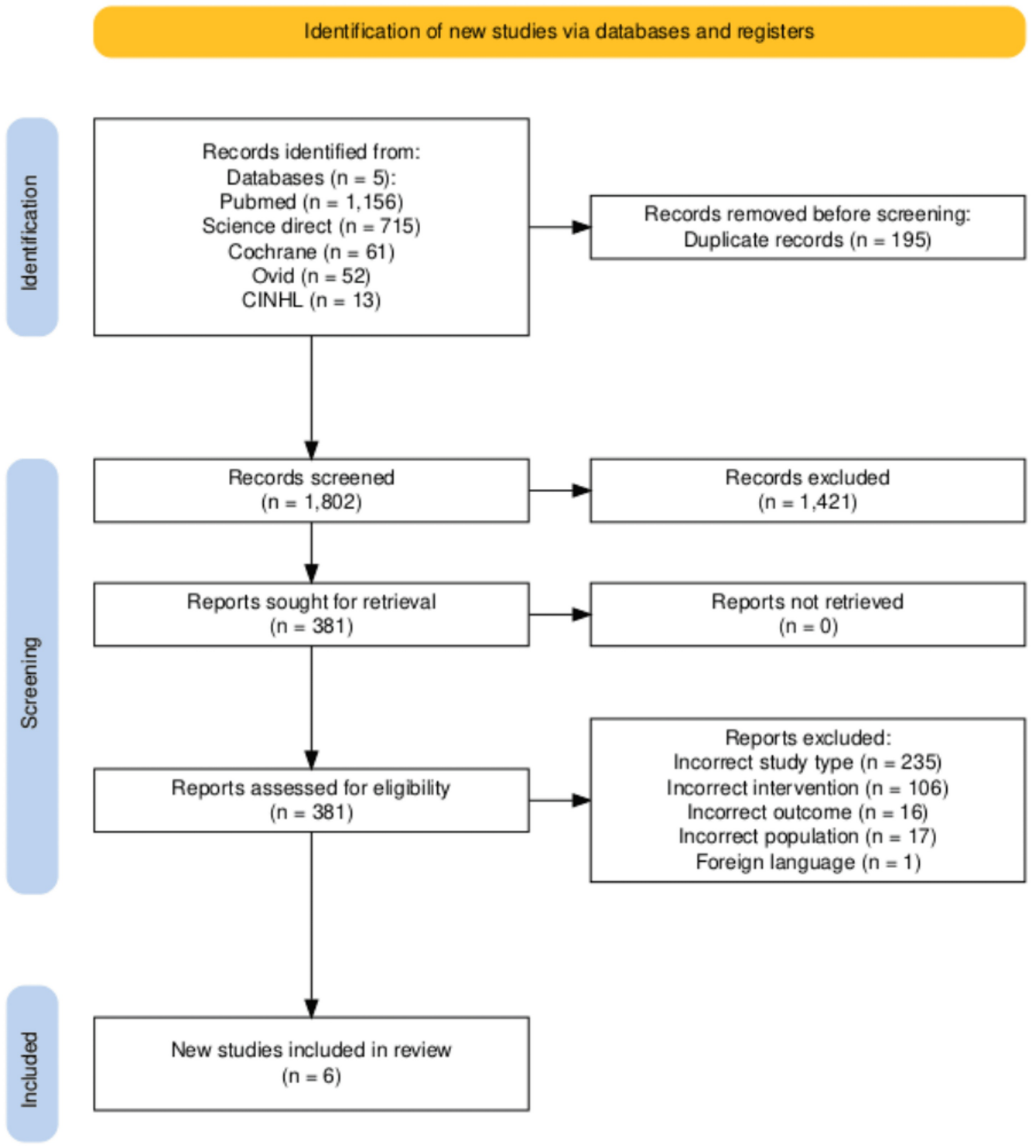
PRISMA flow diagram of study selection

A total of 1,997 records were identified through database searches. After removing 195 duplicates, 1,802 titles and abstracts were screened for eligibility. Following a full-text review of 381 articles, six randomised controlled trials (RCTs) met the inclusion criteria and were included in the final synthesis (Figure 1).

Study Characteristics

Six randomised controlled trials published between 2004 and 2025 were included, enrolling a combined total of 922 participants across diverse cardiac rehabilitation settings in India [23,24], China [25-27], and the United Kingdom [15]. All trials employed a parallel-group RCT design, comparing nurse-led cardiac rehabilitation programmes with routine or standard care. Follow-up ranged from 3 months to 7 months. An overview of the study characteristics, including population, intervention type, and follow-up duration, is presented in Table 2.

**Table 2:** Details of included studies RCT: Randomised Controlled Trial; EF: Ejection Fraction; HF: Heart Failure; NYHA: New York Heart Association; MI: Myocardial Infarction; CVD: Cardiovascular Disease; PCI: Percutaneous Coronary Intervention; CR: Cardiac Rehabilitation; SMS: Short Message Service; NeCR: Nurse-led eHealth Cardiac Rehabilitation; SF-36 V2: Short Form (36) Health Survey, Version 2; MLHFQ: Minnesota Living with Heart Failure Questionnaire; EuroQol VAS: Visual Analogue Scale; QoL: Quality of Life; 6MWT: 6-Minute Walk Test; Borg RPE: Rating of Perceived Exertion; SAQ: Seattle Angina Questionnaire; TUG: Timed Up and Go Test; 6MWD: 6-Minute Walk Distance; ISWT: Incremental Shuttle Walk Test; STS: Sit-to-Stand Test; DASI: Duke Activity Status Index; CHD: Coronary Heart Disease

_Study ID_	_Year_	_Country_	_Study Design_	_Sample Size (n)_	_Population _	_Nurse-Led Intervention Format_	_Control Format_	_Setting (In-person / Tele / Hybrid)_	_Duration of Follow-up_	_Outcomes Extracted_
_Arjunan et al [23]_	_2021_	_India_	_RCT_	_200_	_Hospitalised HF patients Age >31, EF <40%, NYHA4 II–III_	_1:1 education sessions on HF, diet, lifestyle, medication adherence, and symptom recognition; education booklet at discharge_	_Routine care + patient education booklet_	_In Person_	_3 Months_	_QoL (SF-36 V2, MLHFQ)_
_Austin et al [15]_	_2004_	_UK_	_RCT_	_200_	_Age ≥60 with HF diagnosis NYHA II–III, EF ≤40% confirmed by echo_	_In addition to standard care, 8-week nurse-led CR: twice-weekly 2.5h hospital classes and 16-week weekly community exercise_	_Standard care: eight weekly outpatient visits under cardiologist supervision, with monitoring of clinical status by nurse specialist (performance, fluid status, rhythm, labs)_	_In Person_	_6 Months_	_QoL (MLHF, EuroQol, EuroQol-VAS), functional Capacity (6MWT, Borg RPE, NYHA class), hospital re-admission rate and mortality rate_
_Zhang et al [25]_	_2017_	_China_	_RCT_	_199 _	_Patients with angina or prior MI Urban residents, literate in Chinese_	_Nurse-led transitional care programme in addition to routine care. 2 phases of care which involved health education, lectures, home visits, group activities_	_Nurse-led routine care_	_In Person_	_7 Months_	_QoL (SAQ), functional capacity (questionnaire) and hospital re-admission rate_
_Li et al [26]_	_2015_	_China_	_RCT_	_77_	_Older, retired patients who had served in the Army Stable CVD, no cognitive impairment, hospitalised >10 days_	_Nurse-led transitional care program, which included individualised discharge planning, health education, and post-discharge telephone follow-ups_	_Usual care (continue usual activities, pedometer, exercise diary, info booklet)_	_Hybrid_	_12 weeks post discharge_	_QoL (SF-36) and functional Capacity (chair stands, arm curls, TUG, 6MWD, flexibility)_
_Menezes et al [24]_	_2025_	_India_	_RCT_	_100_	_Female cardiac patients from middle income country, age 40–65, stable CVD6 diagnosed for at least 1 month, smartphone literate, Kannada and/or English literate_	_6 month virtual CR via app, website, SMS, WhatsApp, bi-weekly nurse calls; focus on heart-healthy behaviours and secondary prevention_	_Routine cardiologist care_	_Virtual_	_6 months_	_QoL (MacNew score), functional capacity (ISWT, STS, DASI), Hospital re-admission rate, morbidity events and mortality rate_
_Su et al [27]_	_2021_	_China_	_RCT_	_146_	_CHD post-PCI, computer/smartphone literate, no physical activity restrictions, literate in Chinese, primary education or above_	_2 phased NeCR programme involved two phases: goal-setting in hospital, personalised action plans, e-platform, pedometer, peer support, weekly nurse monitoring, feedback, motivational messages_	_Usual care - 10 min didactic session on medication usage and lifestyle changes delivered by staff nurses _	_Hybrid_	_12 weeks_	_QoL (MacNew score), hospital re-admission rate and morbidity events_

Participants generally comprised adults with established cardiovascular disease eligible for secondary prevention through cardiac rehabilitation. Two trials specifically enrolled patients with heart failure. The Indian study Arjunan et al. [23] recruited hospitalised adults aged over 31 years with reduced left ventricular ejection fraction (< 40%) and New York Heart Association (NYHA) class II-III symptoms, while the UK trial Austin et al. [15] included older adults aged 60 years and above with confirmed left ventricular dysfunction (ejection fraction ≤ 40%) verified by echocardiography.

Three studies conducted in China [25-27] focused on patients recovering from coronary heart disease. Zhang et al. [25] enrolled adults with previous myocardial infarction or angina living in urban areas; Li et al. [26] studied retired army veterans hospitalised for stable cardiovascular disease without cognitive impairment. Meanwhile, Su et al. [27] recruited post-percutaneous coronary intervention (PCI) patients who were literate, physically independent, and capable of using smartphone-based rehabilitation tools.

The remaining study, conducted in India by Menezes et al. [24], focused exclusively on female participants aged 40-65 years with stable cardiovascular disease of at least one month’s duration, all of whom were literate and smartphone proficient.

Collectively, the included trials represented a broad spectrum of stable cardiac populations, predominantly middle-aged to older adults with preserved functional capacity and without major comorbidities that would preclude participation in rehabilitation programmes.

Interventions differed in structure and delivery mode but shared common components of education, lifestyle modification, self-management support, and ongoing nurse follow-up. Two studies, Arjunan et al. [23] and Austin et al. [15], implemented centre-based, in-person programmes, combining one-to-one or group education with supervised exercise and counselling over three to six months. Other studies (Zhang et al. [25], Li et al. [26] and Su et al. [27]) evaluated transitional or hybrid models linking hospital discharge education with home- or community-based follow-up. These included structured education sessions, group activities, home visits, telephone monitoring, or digital components such as personalised action plans, pedometer tracking, and motivational feedback. Menezes et al. [24] examined a fully virtual six-month rehabilitation programme delivered via a mobile application, website, SMS, and WhatsApp, supplemented by bi-weekly nurse telephone calls focusing on secondary prevention and long-term behaviour change. Control groups received routine or cardiologist-led care, typically comprising discharge advice, outpatient follow-up, or brief lifestyle counselling without structured exercise or sustained nurse involvement.

Risk of Bias

**Figure 2:**
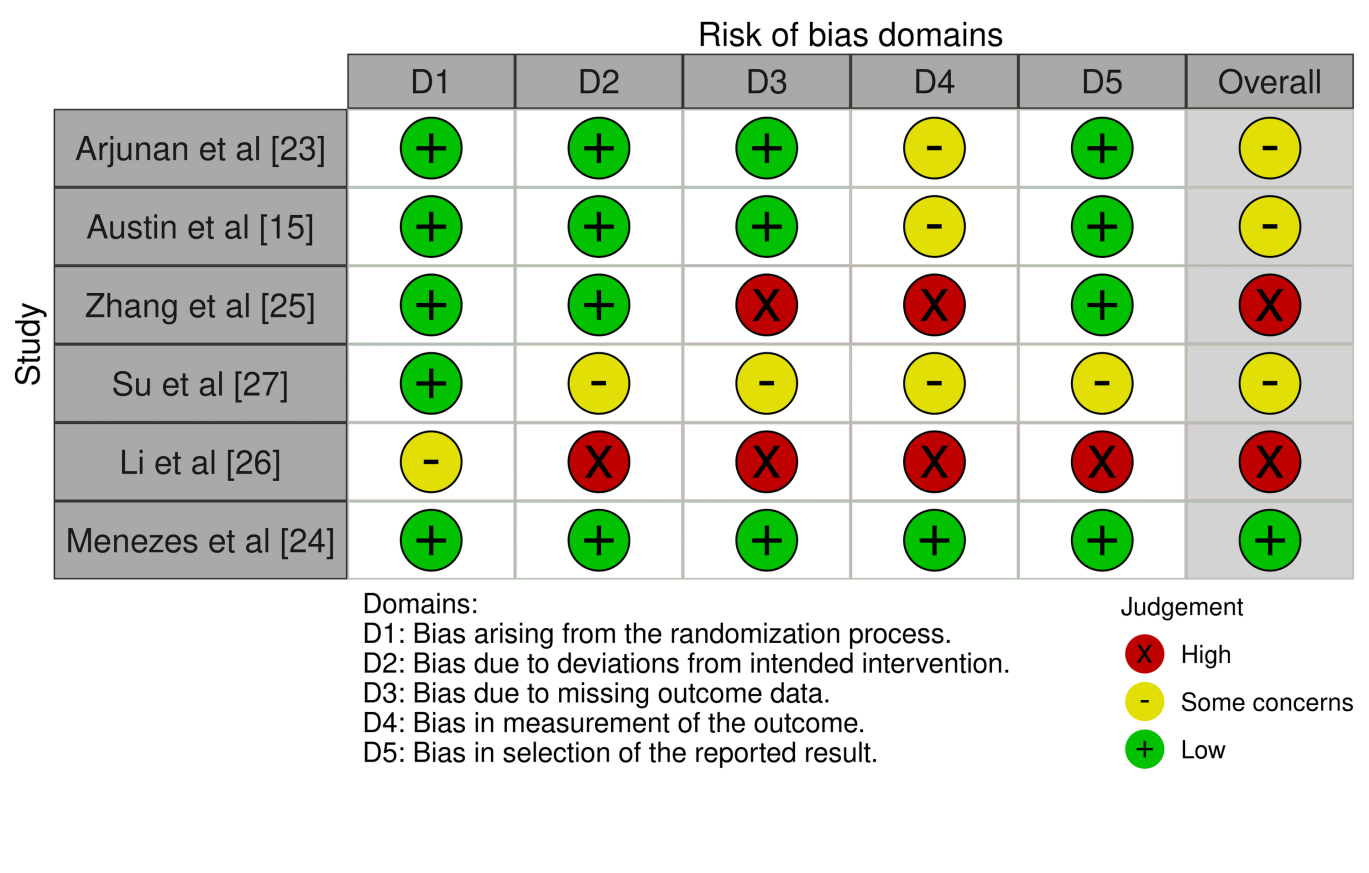
Risk of bias assessment (Cochrane RoB 2 tool) Judgements based on the Cochrane Risk of Bias 2 (RoB 2) tool. “Some concerns” indicates potential bias not sufficient to rate as high, while “high risk” denotes likely bias influencing results.

Based on the Cochrane RoB 2 tool (Figure 2), three studies (Arjunan et al. [23], Austin et al. [15], and Su et al. [27]) showed some concerns, two studies (Zhang et al. [25] and Li et al. [26]) were at high risk, and one (Menezes et al. [24]) was low risk overall. The main sources of bias were missing outcome data and the measurement of outcomes.

Findings

The study results and outcomes are summarised in Table 3, with detailed data for each individual trial. Findings were grouped according to the outcome domains assessed, and effect sizes and clinical significance are also presented.

**Table 3:** Results of included studies 6MWD: Six-minute walk distance. 6MWT: Six-minute walk test. CI: Confidence interval. CR: Cardiac rehabilitation. DASI: Duke Activity Status Index. ED: Emergency department. EQ-VAS: EuroQol Visual Analogue Scale. MLHFQ: Minnesota Living with Heart Failure Questionnaire. NeCR: Nurse-executed cardiac rehabilitation. ns: Not significant. NYHA: New York Heart Association. PCI: Percutaneous coronary intervention. QoL: Quality of life. RCT: Randomised controlled trial. RPE: Rating of perceived exertion. SAQ: Seattle Angina Questionnaire. SF-36: Short Form-36 Health Survey. TUG: Timed Up and Go test.

Study	Outcome Domain	Results	Summary of Findings	
Arjunan et al [23]	Quality of Life	SF-36 Physical: ↑ 1.66 points (95% CI 0.25–3.07), p = .02; SF-36 Mental: ↑ 11.05 points, p < .001; MLHFQ Disease-specific: ↑ 10.09 points, p < .001	Significant improvements at Posttest 2 in physical component, mental component, and disease-specific QoL for the intervention group vs control. Disease-specific QoL also improved significantly.	
				Austin et al [15]	Quality of Life	MLHF Physical score ↓ 8.9 points (21.5 [95% CI 19.1–23.9] → 12.6 [95% CI 10.7–14.5]), p < .01; Control ↓ 3.8 points (24.2 [21.8–26.6] → 20.4 [17.8–23.0]), p < .01. MLHF Emotional score ↓ 4.2 points (8.6 [7.1–10.1] → 4.4 [3.4–5.3]), p < .01; Control ↓ 1.9 points (9.9 [8.3–11.5] → 8.0 [6.6–9.4]), p < .05. MLHF Total score ↓ 18.1 points (41.0 [36.0–46.0] → 22.9 [19.5–26.4]), p < .01; Control ↓ 7.4 points (44.3 [39.5–49.1] → 36.9 [32.2–41.6]), p < .05. EuroQol utility score ↑ 0.11 (0.67 [0.62–0.72] → 0.78 [0.75–0.81]), p < .001; Control no significant change (0.65 [0.61–0.70] → 0.65 [0.59–0.71]). EuroQol-VAS ↑ 9.8 points (60.8 [57.0–64.6] → 70.6 [67.3–73.8]), p < .001; Control no significant change (57.4 [53.7–61.2] → 58.4 [54.6–62.1]).	Patients in the intervention group (multidisciplinary CR programme) had significant improvements in disease-specific QoL (MLHF total, physical and emotional subscales) at both 8 and 24 weeks compared with standard care. Generic QoL (EuroQol, EuroQol-VAS) also improved significantly at 24 weeks in the intervention group, whereas the control group showed little change.	
	Functional Capacity	6MWT distance ↑ 44.9 m (275.5 [95% CI 254.1–298.8] → 320.4 [298.5–342.2]), p < .001; control ↓ 6.7 m (259.4 [236.3–282.5] → 252.7 [226.8–278.5]), ns. Borg RPE (post-test) ↓ from 2/2 to 2/1, p < .05; control worsened from 2/2 to 3/2, p < .05. NYHA class improved from 2.44 (0.50) to 2.01 (0.55), p < .001; control no significant change (2.53 → 2.48).	Participants in the intervention group demonstrated significant improvements in functional capacity compared with standard care. At 24 weeks, 92% of patients in the rehabilitation group increased their 6-minute walk test (6MWT) distance, while the control group showed a slight decline. Ratings of perceived exertion (Borg RPE) improved in the intervention group but worsened in the control group. NYHA class also improved significantly in the intervention group at both 8 and 24 weeks, with 45% of patients improving their symptomatic status compared with only 11% in the standard care group.	
	Mortality	Mortality ↑ 1 (Intervention 5 vs. Control 4), ns.	Mortality rates were similar between groups over 24 weeks, with overall deaths less than 6%.	
	Hospitalisations	Hospital admissions ↓ 9.6% (Intervention 10.6% vs. Control 20.2%), p = 0.2, ns. Total admissions ↓ 22 (Intervention 11 vs. Control 33), p < 0.01. Hospital days ↓ 146 days (Intervention 41 vs. Control 187), p < 0.001.	The intervention group experienced fewer hospital admissions, fewer multiple admissions, and significantly fewer days spent in hospital compared with the standard care group.	
Zhang et al [25]	Quality of Life	Intervention group: ↑ 10.2 points (48.1 → 58.3), p < .001 Control group: ↑ 5.6 points (42.9 → 48.5), p < .001 Between-group difference: favours intervention, p < .001	Participants in the nurse-led transitional care programme demonstrated significant improvements in quality of life, as measured by the Seattle Angina Questionnaire (SAQ), compared with routine care. Improvements were consistent over the 7-month follow-up.	
	Functional Capacity	Angina frequency ↑ 26.2 points (35.7 → 61.9) in the intervention group, p < .001; Control group ↑ 13.4 points (34.9 → 48.3), p < .001 Between-group difference: favours intervention, p = .004 Angina stability ↑ 11.0 points (66.2 → 77.2) in the intervention group, p < .001; Control group ↑ 3.1 points (59.2 → 62.3), p = .010 Between-group difference: favours intervention, p < .001 Physical limitations ↑ 3.7 points (71.3 → 75.0) in the intervention group, p < .001; Control group → 70.9 (71.3 → 70.9), ns Between-group difference: not significant, p = .209	The intervention group experienced significant improvements in functional status compared with routine care. Patients reported better angina stability and reduced angina frequency. There was also a trend towards less physical limitation, although this did not reach statistical significance.	
	Hospitalisations	Readmission ↓ 8.2% (Intervention 9.0% → Control 17.2%), p = .087, ns	Patients in the transitional care group had lower hospital readmission rates at 7 months compared with routine care, although the difference did not reach statistical significance.	
Li et al [26]	Quality of Life	Physical functioning: ↑ 2.9 points (64.8 → 67.7) in intervention vs ↑ 0.3 points (66.9 → 67.2) in control, interaction p = .02 Role-physical: ↑ 17.2 points (35.9 → 53.1) in intervention vs ↑ 4.4 points (35.3 → 39.7) in control, interaction p = .04 Bodily pain: ↑ 7.8 points (59.6 → 67.4) in intervention vs ↑ 2.5 points (61.3 → 63.8) in control, interaction p = .02 Vitality: ↑ 4.8 points (71.6 → 76.4) in intervention vs ↑ 1.5 points (71.4 → 72.9) in control, interaction p = .04 Other SF-36 domains: no significant group × time effects	Older adults who participated in the 12-week, home-based exercise programme reported significant improvements in several physical domains of health-related quality of life (SF-36), including physical functioning, role-physical, bodily pain, and vitality. No significant changes were observed in general health, social functioning, role-emotional, or mental health compared with controls.	
	Functional Capacity	Chair stands: ↑ 1.7 reps (10.6 → 12.3) in intervention vs ↑ 0.4 reps (10.9 → 11.3) in control, interaction p < .001 Arm curls: ↑ 1.0 reps (15.2 → 16.2) in intervention vs ↑ 0.3 reps (15.7 → 16.0) in control, interaction p = .02 Timed Up and Go (TUG): ↓ 0.8 s (9.9 → 9.1) in intervention vs ↓ 0.3 s (10.0 → 9.7) in control, interaction p < .001 Six-minute walk distance (6MWD): ↑ 9.8 m (337.8 → 347.6) in intervention vs ↑ 4.4 m (333.9 → 338.3) in control, interaction p = .04 Flexibility (back scratch & chair sit-and-reach): no significant differences	The 12-week, home-based exercise programme significantly improved multiple measures of functional capacity compared with routine care. Intervention participants showed greater gains in lower and upper body strength, walking endurance, and mobility performance. No significant differences were found for flexibility measures.	
Menezes et al [24]	Quality of Life	Quality of Life (MacNew score) Global score: ↑ 5.4 → 5.9 intervention vs ↔ 5.5 → 5.5 control (p < .001) Physical subscale: ↑ 5.4 → 6.0 intervention vs 5.4 → 5.5 control (p < .001) Emotional subscale: ↑ 5.5 → 6.1 intervention vs 5.7 → 5.6 control (p < .001) Social subscale: ↑ 5.3 → 5.7 intervention vs 5.4 → 5.3 control (p < .001)	Women in the TaCT eCR intervention reported significant improvements in overall quality of life as well as across all MacNew subdomains (physical, emotional, social) compared with standard care.	
	Functional Capacity	ISWT distance: ↑ 180 → 200 m intervention vs 145 → 160 m control (p = .002) DASI score: ↑ 5.9 → 7.7 intervention vs 5.0 → 5.6 control (p < .001) Sit-to-Stand: ↑ 18 → 21 intervention vs ↔ 17.5 → 17.5 control (p = .003)	The TaCT eCR programme significantly improved functional capacity compared with routine care, as shown by gains in walking distance, self-reported activity levels, and sit-to-stand performance.	
	Hospitalisations	8.9% (4/45) in the intervention group vs 18.0% (9/50) in control; no multiple events reported.	The intervention group experienced fewer events (ED visits, hospitalisations and cardiac events/procedures) compared with standard care, though the trial was underpowered for statistical testing.	
	Mortality	1 death in the intervention group; none in the control group.	Mortality was rare across both groups and not significantly different between interventions.	
Su et al [27]	Quality of life	Physical QoL: ↑ 0.45 points (95% CI 0.003–0.89), p = 0.049, Hedges’ g = 0.40 Social QoL: ↑ 0.54 points (95% CI 0.07–1.02), p = 0.025, Hedges’ g = 0.41 Global QoL: ↑ 0.56 points (95% CI 0.25–0.87), p < 0.001 (Bonferroni-adjusted) Emotional QoL: no significant change (p = 0.133)	The NeCR intervention did not significantly improve overall MacNew QoL scores at first analysis, but significant between-group improvements were observed in the physical and social subdomains at 12 weeks. After Bonferroni adjustment for multiple testing, the global MacNew score at 12 weeks also reached statistical significance, confirming overall QoL benefit. No significant differences were found in the emotional subdomain.	
	Hospitalisations	Re-hospitalisations (intervention): 2 participants (1 underwent PCI) Clinical events (control): 4 participants – 2 ED visits for tachy/bradycardia, 2 re-hospitalisations requiring PCI Time to hospital admission: slightly longer in intervention group; not significant (p = 0.25)	Unplanned health service use (hospitalisation, ED visits, PCI) was lower in the NeCR group compared with usual care, although differences were not statistically significant.	

Overall Quality of Life

Across six randomised controlled trials, nurse-led cardiac rehabilitation (CR) consistently improved overall quality of life compared with routine care. Benefits were observed across both general and disease-specific instruments, including the Minnesota Living with Heart Failure Questionnaire (MLHFQ), SF-36, EQ-5D, MacNew, and Seattle Angina Questionnaire (SAQ). Most studies reported changes that exceeded clinically significant differences.

Arjunan et al. [23] demonstrated significant gains in SF-36 physical (+1.66) and mental (+11.05) domains, alongside disease-specific QoL improvements (+10.09; p < .001). Similarly, Austin et al. [15] reported substantial improvements in MLHFQ total score, decreasing by 18.1 points (from 41.0 [36.0-46.0] to 22.9 [19.5-26.4]; p < .01), compared with a smaller reduction in controls (↓ 7.4 points; from 44.3 [39.5-49.1] to 36.9 [32.2-41.6]; p < .05). Physical and emotional subscales also improved significantly in the intervention group-physical: ↓ 8.9 points (from 21.5 [19.1-23.9] to 12.6 [10.7-14.5]; p < .01); emotional: ↓ 4.2 points (from 8.6 [7.1-10.1] to 4.4 [3.4-5.3]; p < .01) versus smaller changes in controls (↓ 3.8 and ↓ 1.9 points, respectively). Generic QoL followed the same trend. EQ-5D utility increased by 0.11 (from 0.67 [0.62-0.72] to 0.78 [0.75-0.81]; p < .001), and EQ-VAS rose by 9.8 points (from 60.8 [57.0-64.6] to 70.6 [67.3-73.8]; p < .001), while controls showed no significant change.

Zhang et al. [25] reported a 10.2-point improvement in SAQ overall score for the intervention group (from 48.1 to 58.3; p < .001), compared with a 5.6-point increase in controls (from 42.9 to 48.5; p < .001), with the between-group difference significantly favouring the nurse-led programme (p < .001).

Li et al. [26] observed notable gains across several SF-36 physical domains, including physical functioning (+2.9 vs. +0.3; p = .02), role-physical (+17.2 vs. +4.4; p = .04), bodily pain (+7.8 vs. +2.5; p = .02), and vitality (+4.8 vs. +1.5; p = .04), while other domains showed no significant interaction effects. Menezes et al. [24] demonstrated significant improvements in overall quality of life, with the MacNew global score increasing from 5.4 to 5.9 in the intervention group versus no change in controls (5.5 to 5.5; p < .001). All MacNew subdomains improved physical (5.4 to 6.0), emotional (5.5 to 6.1), and social (5.3 to 5.7), each reaching statistical significance (p < .001).

Su et al. [27] reported comparable findings, with the nurse-led e-CR group showing significant gains in MacNew global (+0.56 [95% CI 0.25-0.87]; p < .001), physical (+0.45 [95% CI 0.003-0.89]; p = .049), and social (+0.54 [95% CI 0.07-1.02]; p = .025) domains, while emotional QoL remained unchanged (p = .133).

Taken together, these findings support a consistent benefit across diverse instruments and populations. Improvements were evident within 8-12 weeks and sustained for up to seven months. According to the GRADE assessment, the certainty of evidence was rated moderate, downgraded one level for risk of bias due to self-reported measures and modest sample sizes but supported by the uniform direction and magnitude of benefit across all studies.

Functional Capacity

Improvements in functional capacity were consistently reported across trials [15,24-26], favouring nurse-led cardiac rehabilitation (CR) over routine care. Outcomes were assessed using validated objective measures such as the 6-minute walk test (6MWT) and incremental shuttle walk test (ISWT), along with strength, mobility, and endurance assessments, and patient-reported tools including the Borg Rating of Perceived Exertion (RPE) and Duke Activity Status Index (DASI).

Austin et al. [15] reported a significant increase in 6MWT distance of +44.9 m (275.5 [95% CI 254.1-298.8] → 320.4 [298.5-342.2]; p < .001) compared with a non-significant 6.7 m decline in controls (259.4 [236.3-282.5] → 252.7 [226.8-278.5]). Perceived exertion improved in the intervention group (Borg RPE 2/2 → 2/1; p < .05), while controls worsened (2/2 → 3/2; p < .05). NYHA class also improved significantly (2.44 → 2.01; p < .001), with no change in controls (2.53 → 2.48).

Zhang et al. [25] observed similar benefits, with significant improvements in angina frequency (+26.2 points [35.7 → 61.9]; p < .001) and angina stability (+11.0 points [66.2 → 77.2]; p < .001) compared with smaller gains in controls (+13.4 [34.9 → 48.3]; p < .001 and +3.1 [59.2 → 62.3]; p = .010, respectively). Between-group differences favoured the nurse-led programme (p = .004 and p < .001). Physical limitation scores improved by +3.7 points (71.3 → 75.0; p < .001) but did not reach statistical significance between groups (p = .209).

Li et al. [26] found significant gains across several performance domains. Chair-stand repetitions increased by +1.7 (10.6 → 12.3) vs. +0.4 (10.9 → 11.3) in controls (p < .001), arm curls by +1.0 (15.2 → 16.2) vs. +0.3 (15.7 → 16.0) (p = .02), and mobility improved with a 0.8 s reduction in Timed Up and Go (TUG) (9.9 → 9.1) vs. 0.3 s (10.0 → 9.7) (p < .001). 6MWT distance rose +9.8 m (337.8 → 347.6) vs. +4.4 m (333.9 → 338.3) (p = .04), while flexibility measures showed no difference.

Menezes et al. [24] further demonstrated improved exercise capacity and daily activity performance. ISWT distance increased from 180 m to 200 m vs. 145 m to 160 m in controls (p = .002), DASI scores rose 5.9 → 7.7 vs. 5.0 → 5.6 (p < .001), and sit-to-stand repetitions improved 18 → 21 vs. no change (17.5 → 17.5; p = .003).

Collectively, these findings show consistent, statistically significant, and clinically meaningful gains in physical endurance, mobility, and functional performance following nurse-led CR compared with routine care. Evidence certainty was rated moderate, downgraded for minor risk of bias but strengthened by the uniform direction and magnitude of benefit across all included studies.

Hospitalisations

Four studies [15,24-25,27] evaluated hospital utilisation outcomes, with most showing reductions in readmission frequency and hospital stay duration among participants in nurse-led cardiac rehabilitation (CR) compared with routine care.

Austin et al. [15] reported a 9.6% absolute reduction in hospital admissions (10.6% vs. 20.2%; p = 0.2, ns) and a significant decrease in total admissions (11 vs. 33; p < 0.01). The intervention group also experienced a marked reduction in cumulative hospital days, spending 146 fewer days in hospital overall compared with controls (41 vs. 187 days; p < 0.001).

Zhang et al. [25] found an 8.2% reduction in hospital readmissions (9.0% vs. 17.2%; p = .087), indicating a favourable trend toward fewer hospitalisations in the nurse-led transitional care group, although this did not reach statistical significance.

Menezes et al. [24] similarly reported fewer events in the nurse-led cohort, with 8.9% (4/45) of participants experiencing hospital readmissions compared with 18.0% (9/50) in controls. No multiple or recurrent hospitalisations were reported.

Su et al. [27] observed fewer unplanned hospital encounters in the intervention group, with two participants hospitalised (one undergoing PCI) compared with four in the control group (two emergency visits for tachycardia/bradycardia and two requiring PCI). Time to hospital admission was slightly longer in the intervention group but not statistically significant (p = 0.25).

Collectively, these findings demonstrate a consistent reduction in hospital utilisation among participants receiving nurse-led CR, particularly in overall admissions and length of stay, though some individual trials lacked statistical power to confirm significance due to low event rates. According to the GRADE Summary of Findings table, the certainty of evidence for hospital readmissions was rated low and downgraded for risk of bias and imprecision due to limited event frequency and small sample sizes across studies.

Mortality

Two studies [15,24] reported mortality outcomes, with no evidence of significant differences between nurse-led cardiac rehabilitation (CR) and routine care.

Austin et al. [15] observed comparable mortality rates between groups, with five deaths in the intervention group versus four in the control group (ns). Similarly, Menezes et al. [24] reported one death in the intervention group and none in the control group.

Across both trials, overall mortality rates were low (<3%) and statistically nonsignificant, with no indication of harm associated with nurse-led CR. According to the GRADE Summary of Findings table, the certainty of evidence for mortality was rated very low, downgraded for risk of bias, imprecision, and the very small number of events observed within short follow-up periods (<6 months).

Certainty of Evidence

The overall certainty of evidence ranged from moderate to very low across the included outcomes. Evidence for quality of life and functional capacity was moderate, indicating consistent and clinically meaningful improvements with nurse-led cardiac rehabilitation compared to routine care. Hospital readmissions were supported by low-certainty evidence, suggesting a possible reduction in events, while mortality was based on very low-certainty evidence, showing no clear difference between groups. Full explanations for certainty ratings are provided below in Table 4.

**Table 4:** GRADE assessment table Explanations a. QoL was assessed using self-reported questionnaires across all trials, which are subject to reporting bias. Some trials also had modest samples and concerns in conduct and reporting, reducing confidence in the estimates b. Downgraded one level for risk of bias: several outcomes were self-reported (DASI, SAQ functional status), sample sizes were modest in some trials, and there were concerns in trial conduct/reporting and handling of missing data, which reduce confidence in the estimates. c. Downgraded one level for risk of bias: Readmission outcomes were not always systematically defined or consistently reported (e.g., combined morbidity outcomes, unclear ascertainment, or lack of statistical testing). Several trials were relatively small for detecting differences in such infrequent events, meaning a few cases could substantially change results. In addition, missing data handling and selective emphasis on favourable findings were not always transparent. d. few events overall, wide uncertainty, underpowered to detect meaningful differences. e. Downgraded one level for risk of bias: Mortality was not a prespecified outcome, was inconsistently reported (grouped with morbidity events in one trial), and was not statistically analysed in Menezes f. Downgraded two levels for imprecision: Very few events were observed. The short follow-up periods (≤6 months) further limit confidence, as mortality differences may not emerge until longer-term follow-up. The true effect could therefore include benefit, no effect, or harm.

Certainty assessment							No of patients		Effect		Certainty	Importance
No of studies	Study design	Risk of bias	Inconsistency	Indirectness	Imprecision	Other considerations	Nurse-led cardiac rehabilitation programme	Routine care	Relative (95% CI)	Absolute (95% CI)		
Quality of Life (follow-up: mean 5 months; assessed with: MLHFQ, Euro-QoL, SAQ, SF-36, MacNew QoL)												
5	randomised trials	serious^a^	not serious	not serious	not serious	none	Nurse-Led Cardiac Rehabilitation Program Participants = 364, Routine Care Participants = 369. All five RCTs reported improvements in overall quality of life with nurse-led cardiac rehabilitation compared to routine care. Effects were consistent across different validated tools (MLHFQ, EuroQol, SAQ, MacNew), often exceeding minimal clinically important differences. Benefits emerged as early as 8–12 weeks and were sustained up to 7 months.				⨁⨁⨁◯ Moderate^a^	CRITICAL
Functional Capacity (assessed with: 6MWT, Borg RPE, SRAHP, Senior Fitness Test, ISWT, DASI)												
4	randomised trials	serious^b^	not serious	not serious	not serious	none	Nurse-Led Cardiac Rehabilitation Program Participants = 267, Routine Care Participants = 272. All four RCTs assessed functional capacity and reported improvements with nurse-led CR compared to routine care. Objective tests showed significant gains in 6MWT, ISWT, mobility (TUG), and strength (Sit to Stand reps and chair stands). Self-reported activity also improved (DASI, SAQ frequency and stability). One trial found no significant between group difference in SAQ physical limitation, but the overall direction of effect consistently favoured intervention. Most gains were clinically meaningful and sustained up to 6–7 months.				⨁⨁⨁◯ Moderate^b^	CRITICAL
Hospital Re-Admissions												
4	randomised trials	serious^c^	not serious	not serious	serious^d^	none	Nurse-Led Cardiac Rehabilitation Program Participants = 264, Routine Care Participants = 272 Four RCTs (N = 539) showed fewer hospital readmissions with nurse-led CR compared to routine care, though most were not statistically significant due to low event rates.				⨁⨁◯◯ Low^c,d^	CRITICAL
Mortality												
2	randomised trials	serious^e^	not serious	not serious	very serious^f^	none	Nurse-Led Cardiac Rehabilitation Program Participants = 135, Routine Care Participants = 144. Very few deaths occurred in both studies. Mortality rates were similar between nurse-led CR and routine care, with no evidence of benefit or harm.				⨁◯◯◯ Very low^e,f^	CRITICAL

Discussion

Principle Findings

This systematic review synthesised evidence from six randomised controlled trials evaluating the effectiveness of nurse-led cardiac rehabilitation (CR) compared with routine care in adults with established cardiovascular disease. Owing to heterogeneity in interventions, measures, and follow-up durations, results were synthesised narratively. The findings demonstrate that nurse-led CR programmes consistently improve quality of life and functional capacity, with favourable trends toward reduced hospital readmissions and shorter length of stay, and no significant difference in mortality. Overall, these findings suggest that nurse-led CR is a safe, effective, and potentially scalable alternative to standard care, providing comparable, if not superior, patient-centred outcomes.

Interpretation and clinical relevance

Quality of Life

The most notable finding of this review is the consistent and meaningful improvement in quality of life (QoL) across all included studies, measured with a range of validated instruments. Improvements were seen not only in overall scores but also across physical, emotional, and social domains and often exceeded established thresholds for clinical significance. For example, Austin et al. [15] reported an 18-point reduction in the MLHFQ total score, while Zhang et al. [25] found a 10-point improvement in the SAQ, which are changes that are clearly perceptible to patients in their daily lives [15, 24]. These benefits were also evident in virtual settings, with significant improvements in MacNew global and subdomain scores observed by Menezes et al .[24] and Su et al. [27]. These findings imply that the benefits arise primarily from the principles of nursing care, holistic, continuous, patient-centred support, rather than the delivery format, effectively tackling the psychosocial and educational challenges of managing chronic illness.

Functional Capacity

These QoL improvements are strongly supported by objective and patient-reported gains in functional capacity. The 44-metre improvement in the 6-minute walk test reported by Austin et al. [15] is not only statistically significant but is associated with a reduced risk of mortality in heart failure populations. This finding is reinforced by significant improvements in walking endurance in other trials (Li et al. [26] and Menezes et al. [24]) and improved performance in strength and mobility tasks (e.g., chair stands and the Timed Up-and-Go test). Additionally, reductions in angina frequency and improved symptom stability reported by Zhang et al. [25] directly translate into greater ability to perform daily activities. Together, these improvements in physical function and symptom burden create a positive feedback cycle, promoting independence, self-efficacy, and the emotional well-being reflected in QoL measures.

Hospitalisations and Mortality

Although reductions in hospital readmissions did not always reach statistical significance in individual trials, the consistent trend across all four studies reporting this outcome suggests a meaningful real-world impact. The significant reduction in total hospital days observed by Austin et al. [15] is particularly noteworthy, indicating that nurse-led care may reduce the severity of exacerbations. This likely reflects improved self-management, medication adherence, and earlier symptom recognition, which are core components of the nurse-led interventions. Low event rates and relatively short follow-up in these trials could have led to imprecision, highlighting the need for larger, longer-term studies to confirm these effects.

Importantly, the absence of any signal for harm, with mortality rates similar across groups [15,27], highlights the safety of nurse-led CR models, a key consideration for clinicians and policymakers. However, the very low certainty of evidence for mortality, largely due to the rarity of events, emphasises the need for longer-term research designed to detect differences in key clinical outcomes.

Comparison With Previous Literature

The results of this review complement and extend the established evidence base supporting CR as an effective secondary prevention strategy. Consistent with prior Cochrane syntheses [17,18], our findings confirm that the improvements in functional capacity and QoL achieved through routine care are equally attainable when rehabilitation is delivered by nurses. McDonagh et al. (2023) [18] found no significant differences in total mortality or exercise capacity when comparing home-based CR to centre-based, with over 90% of included studies reporting equivalent QoL outcomes. Anderson and Taylor (2014) [17] further consolidated these findings in an overview of six Cochrane reviews, confirming consistent improvements in QoL and reductions in hospital readmissions across CR models. Collectively, these analyses demonstrate that the benefits of CR are robust across settings and formats and are not dependent on physician-led structures. Furthermore, the independent contribution of nurses within cardiac rehabilitation and secondary prevention has been examined in focused systematic reviews. Chiang et al. [28] synthesised 12 randomised controlled trials evaluating nurse-led, patient-centred care models for secondary cardiac prevention in patients with coronary heart disease. Their meta-analyses demonstrated significant short- to medium-term improvements in smoking cessation, adherence to physical activity advice, and optimisation of lipid profiles compared with routine care. Similarly, Stromberg et al. [29] conducted a randomised controlled trial of 106 patients hospitalised with heart failure, comparing structured follow-up in a nurse-led heart failure clinic to routine care. The nurse-led intervention included protocol-guided medication titration, clinical assessment, and tailored patient education with family support. After 12 months, the nurse-led group demonstrated significantly fewer deaths, hospitalisations, and shorter hospital stays. Self-care scores were also significantly higher at 3 and 12 months.

However, a previous review by Mares et al. [19] reported mixed effects of nurse-led CR on QoL in post-CABG populations. Our broader synthesis demonstrates a consistent, positive effect across multiple cardiac conditions, including heart failure, post-MI, and post-PCI. This suggests generalisability of benefits across cardiovascular conditions. Moreover, by including contemporary trials employing hybrid and fully virtual delivery models (Menezes et al. [24] and Su et al. [27]), our review highlights the flexibility and relevance of nurse-led care within modern healthcare systems.

The evolving structure of CR delivery has also been recognised at an international policy level. The International Council of Cardiovascular Prevention and Rehabilitation consensus statement by Grace et al. [30] analysed data from 111 countries and identified nurse-led and hybrid models as essential for scalable, cost-effective expansion of CR, particularly in low- and middle-income settings where cardiologist availability is limited. The statement emphasised nurses’ leadership in risk-factor monitoring, patient education, and remote supervision as core competencies within modern CR services.

Limitations

This review has several important limitations that should be considered when interpreting the findings. First, the outcomes ultimately analysed differed from those originally outlined in the PROSPERO registration. This was primarily due to variations in how outcomes were reported across studies and the limited availability of consistent data for certain prespecified endpoints. Second, a quantitative meta-analysis could not be conducted because of the substantial methodological and clinical heterogeneity between trials. Differences were observed in sample size, study design, follow-up duration, and the instruments used to assess key outcomes, such as quality of life and functional capacity.

Moreover, the included trials lacked uniformity in outcome measures, with multiple validated tools employed across studies, limiting direct comparison and pooled synthesis. Although all interventions were nurse-led, the nature of these programmes varied considerably, encompassing in-person, home-based, hybrid, and online formats. This variation in delivery, frequency, and intensity may have influenced the observed effects and limited the generalisability of the findings. Additional potential sources of bias include the restriction to English language studies, exclusion of grey literature, and instances where data were approximated from published figures when raw data were unavailable, which may introduce minor imprecision. Lastly, the small number of eligible studies, many with modest sample sizes and short follow-up periods, further restricts the ability to draw firm conclusions regarding long-term clinical outcomes such as hospital readmissions and mortality.

## Conclusions

This systematic review provides evidence that nurse-led cardiac rehabilitation programmes can improve quality of life and functional capacity in adults with cardiovascular disease compared to routine care. The benefits were consistent across multiple validated tools and were often clinically meaningful, underscoring the important role of nurses in supporting secondary prevention, education, and patient self-management. Although reductions in hospital readmissions were reported in several studies, these findings were based on low-certainty evidence and were not always statistically significant. Mortality outcomes were infrequent, and the available data were insufficient to determine any clear difference between groups. The strength of these conclusions is limited by the small number of eligible trials, modest sample sizes, short follow-up durations, and heterogeneity in both intervention design and outcome assessment. Variation in programme structure, ranging from in-person to hybrid and online formats, also limits generalisability and precludes quantitative synthesis. Despite these limitations, the consistency of direction of benefit across studies suggests that nurse-led rehabilitation can produce meaningful patient-centred improvements in recovery and wellbeing.

Importantly, several included studies also indicated that nurse-led models of care may be cost-effective, owing to reduced resource utilisation, fewer hospital admissions, and improved long-term adherence to lifestyle and medication strategies. Wider implementation of such programmes could therefore offer both clinical and economic value within healthcare systems facing increasing cardiovascular disease burden and workforce constraints. Future research should prioritise large, multicentre randomised trials with standardised outcome reporting, longer follow-up, and formal economic evaluation to confirm the durability and cost-effectiveness of nurse-led cardiac rehabilitation. Overall, the evidence supports nurse-led cardiac rehabilitation as an effective, adaptable, and potentially sustainable approach that can complement routine care and improve outcomes for patients with cardiovascular disease.
